# New steady-state microbial community compositions and process performances in biogas reactors induced by temperature disturbances

**DOI:** 10.1186/s13068-014-0182-y

**Published:** 2015-01-22

**Authors:** Gang Luo, Davide De Francisci, Panagiotis G Kougias, Treu Laura, Xinyu Zhu, Irini Angelidaki

**Affiliations:** Shanghai Key Laboratory of Atmospheric Particle Pollution and Prevention (LAP3), Department of Environmental Science and Engineering, Fudan University, 200433 Shanghai, China; Department of Environmental Engineering, Technical University of Denmark, DK-2800 Kgs Lyngby Copenhagen, Denmark

**Keywords:** Anaerobic digestion, Microbial community composition, Process performance, Temperature disturbance

## Abstract

**Background:**

The microbial community in a biogas reactor greatly influences the process performance. However, only the effects of deterministic factors (such as temperature and hydraulic retention time (HRT)) on the microbial community and performance have been investigated in biogas reactors. Little is known about the manner in which stochastic factors (for example, stochastic birth, death, colonization, and extinction) and disturbance affect the stable-state microbial community and reactor performances.

**Results:**

In the present study, three replicate biogas reactors treating cattle manure were run to examine the role of stochastic factors and disturbance in shaping microbial communities. In the triplicate biogas reactors with the same inoculum and operational conditions, similar process performances and microbial community profiles were observed under steady-state conditions. This indicated that stochastic factors had a minor role in shaping the profile of the microbial community composition and activity in biogas reactors. On the contrary, temperature disturbance was found to play an important role in the microbial community composition as well as process performance for biogas reactors. Although three different temperature disturbances were applied to each biogas reactor, the increased methane yields (around 10% higher) and decreased volatile fatty acids (VFAs) concentrations at steady state were found in all three reactors after the temperature disturbances. After the temperature disturbance, the biogas reactors were brought back to the original operational conditions; however, new steady-state microbial community profiles were observed in all the biogas reactors.

**Conclusions:**

The present study demonstrated that temperature disturbance, but not stochastic factors, played an important role in shaping the profile of the microbial community composition and activity in biogas reactors. New steady-state microbial community profiles and reactor performances were observed in all the biogas reactors after the temperature disturbance.

**Electronic supplementary material:**

The online version of this article (doi:10.1186/s13068-014-0182-y) contains supplementary material, which is available to authorized users.

## Background

Anaerobic digestion (AD) is widely used in the treatment of organic wastes to achieve reduction of the wastes with simultaneous production of biogas [1]. The production of biogas via AD is a complex process, involving many different microbial species [2]. The complex organic compounds are first hydrolyzed into oligomers and monomers, and then further converted to acetate, CO_2_, and H_2_ by various fermenting bacteria. The methanogenesis is the final step to convert acetate, CO_2_, and H_2_ to CH_4_ by methanogenic archaea. The syntrophic relationship between bacteria and archaea is essential for the stability of the biogas process [3].

It is crucial to understand the processes and factors controlling the microbial community composition in biogas reactors. The mechanisms of community assembly and the critical factors shaping species composition and structure remain controversial in ecology [4-6]. The traditional niche-based theory supports the idea that the community is shaped mainly by deterministic factors such as competition and niche differentiation, and thereby asserts that community composition should converge toward a single pattern under similar environmental conditions [7]. In contrast to niche-based theory, neutral theory assumes that many natural community patterns can be generated under similar environmental conditions by stochastic factors considering birth, death, dispersal, and speciation and disregards the differences between species at the same trophic level [8]. In addition, disturbance was also shown to play an important role in the community assembly since the disturbance could kill or damage certain species and promote the growth of other species that are resistant to the disturbance [9,10].

Microbial community compositions in biogas reactors have been studied for several decades, and deterministic factors including temperature, hydraulic retention time (HRT), and substrate type have been demonstrated to play an important role in shaping microbial communities [11,12]. However, based on the neutral theory, stochastic factors may be important in shaping the highly diverse microbial communities in biogas reactors. Up to now, it is still unknown whether there are different microbial community patterns under the same environmental conditions in biogas reactors if stochastic factors are determining the microbial communities. Although different disturbances (such as temperature and organic loading) on the biogas process have been evaluated before, most of the studies focused only on the reactor performances, and the effect of disturbance on the community assembly was not documented [13-16]. It is still unknown whether the disturbance in the biogas reactors would lead to different steady-state microbial community compositions and reactor performances.

Traditional molecular technologies for microbial community analysis (for example, polymerase chain reaction-denaturing gradient gel electrophoresis, terminal restriction fragment length polymorphism, and cloning) can only identify the most abundant microorganisms in the microbial community. The less abundant yet functionally important microorganisms cannot be detected [17]. Therefore, using traditional molecular technologies, it is difficult to study variations of less abundant microorganisms in different samples. With the newly developed sequencing technologies, it is possible to define microbial community composition with a high sequencing depth. The Ion Torrent Personal Genome Machine (PGM) (Life Technologies), launched in early 2011, has the highest throughput compared with 454 GS Junior (Roche) and Miseq (Illumina), thus making the high-throughput sequencing cost effective and time saving [18].

Based on the above considerations, the objective of this study was to understand the role of stochastic factors (based on neutral theory) and disturbance in the steady-state microbial community assembly and functions in biogas reactors. We ran three replicate biogas reactors treating cattle manure to first determine whether similar microbial communities would be achieved at steady states where the reactors were operated under the same conditions. In most modern biogas plants one attempts to keep a constant temperature, as temperature stability is of outmost importance for the biogas process. Nevertheless, biogas reactors may be subjected to undesired temperature fluctuations due to various technical problems such as heat exchanger or pump failures or fouling in temperature sensors [16,19]. Therefore, temperature disturbance was introduced to the three reactors in order to determine if and to what extent the temperature disturbance would alter the steady-state microbial community. In addition, the reactor performances including biogas production, pH, and total volatile fatty acids (VFAs) were monitored. The microbial community composition was analyzed by Ion Torrent sequencing of 16 s rRNA gene amplicons.

## Results and discussion

### Reactor performance

**Figure 1:**
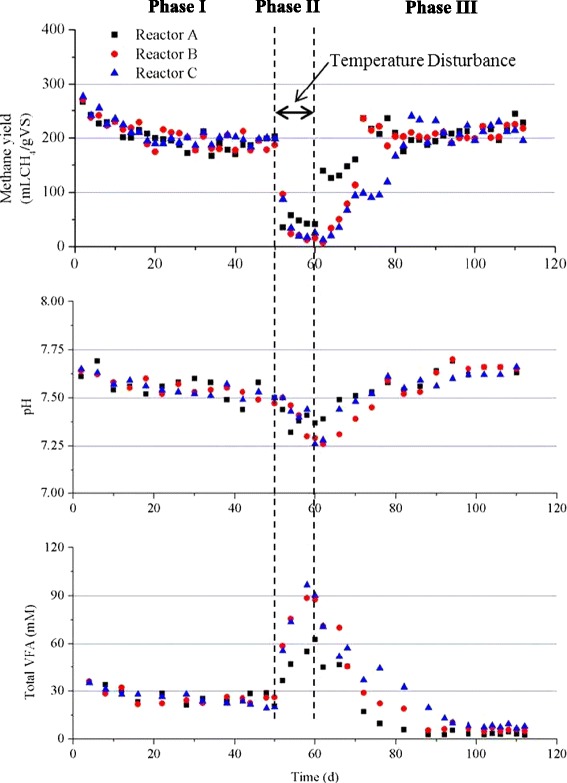
**Reactor performances for the whole operational period.**

**Figure 2:**
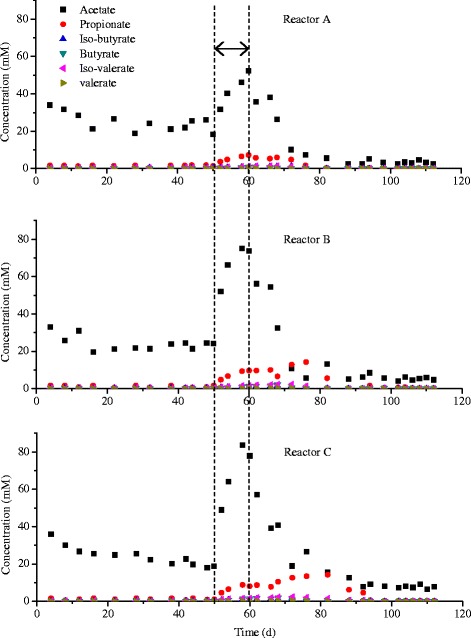
**Individual VFAs changes for the whole operational period.**

The monitoring profiles for methane yield, pH, and total VFAs in the three reactors are shown in Figure 1, and the overall performances of the reactors at steady state are summarized in Table 1. The initial higher methane yield was due to the methane production from the inoculum, since there are still organics in the inoculum which can be digested. After around 30 days’ operation, the methane yields were relatively stable. The steady-state methane yields for the three reactors were not significantly different (*P* < 0.05): 194 ± 7.3, 189 ± 14.5, and 195 ± 6.9 mL/g volatile solids (VS) for reactors A, B, and C, respectively. Both the pH values (around 7.5) and the total VFAs concentrations were also similar for all three reactors. Acetate was the dominant VFAs, as seen in Figure 2. The above results indicated that the replicate reactors (A, B, and C) did not present obvious differences in their performances.

**Table 1:** **Summary of the reactor performances at steady state before (phase I, 0 to 50 days) and after (phase II, 61 to 112 days) temperature disturbance**

**Parameter**	**A**_**b**_	**A**_**a**_	**B**_**b**_	**B**_**a**_	**C**_**b**_	**C**_**a**_
Methane yield (mLCH_4_/gVS)	194 ± 7.3	220 ± 17.5	189 ± 14.5	213 ± 11.7	195 ± 6.9	214 ± 13.1
pH	7.50 ± 0.06	7.64 ± 0.02	7.51 ± 0.04	7.66 ± 0.01	7.52 ± 0.04	7.60 ± 0.02
Total VFAs(mM)	25.5 ± 3.9	3.3 ± 0.9	24.9 ± 1.7	5.3 ± 0.6	21.1 ± 1.9	7.8 ± 1.2

Subscript b means before temperature disturbance, subscript a means after temperature disturbance.

From day 50 (phase II), the temperatures of the reactors were changed (A 25°C, B 45°C, C 55°C) from the original temperature of 37°C. A sharp decrease in methane yields was observed in all the reactors, together with a decrease in pH and an increase in total VFAs. After 10 days at the new temperatures, the total VFAs increased to around 60 mM for reactor A, and to around 90 mM for both reactors B and C, which clearly indicates that the increase of temperature had a more profound effect on the stability of the reactors. There are several reasons leading to the higher VFAs accumulation when the temperatures were increased. It could be due to faster adaptation of acidogenic bacteria or to a greater temperature span of acidogens at higher temperatures compared to methanogens, as they generally grow more slowly and have a narrower temperature span [20,21]. Among the total VFAs, acetate was still the dominant component, although propionate was also accumulated, which is consistent with previous reports that propionate is easy to accumulate when the biogas reactor is disturbed [22,23].

From day 60 (phase III), the operating temperatures of all the reactors were changed back to 37°C. The methane yield increased immediately for reactor A. However, a slower recovery of the methane yield was observed for reactors B and C, which might indicate that the higher temperature disturbances (45°C and 55°C) had a more negative impact on the stability of the biogas reactors. In particular, reactor C took around 10 days before the methane yield increased to a similar level to that of the steady-state level in phase I. The fast increase of methane yield in reactor A was in good agreement with the fast decrease of total VFAs. During the steady states of phase III, the methane yields for reactors A, B, and C were 220 ± 17.5, 213 ± 11.7, and 214 ± 13.1 mL/gVS, respectively, and there were no significant differences (*P* < 0.05) among the reactors. However, the methane yields were all significantly higher (around 10%) than those in phase I. The results indicated that the temperature disturbances affected the performances of all the reactors, although the operational conditions were the same for all the reactors in both phases I and III. The lower VFAs concentrations in phase III compared with those in phase I could explain the increased methane yields. However, the decreased VFAs concentration only accounted for 10% or less of the increased methane yield. This indicates that the increased methane yield could also be related with the increased hydrolysis of the solid part in cattle manure, which would lead to higher methane production. The lower VFAs concentrations in phase III for all the three reactors also resulted in the relatively higher pH (around 7.6).

### Microbial community analysis

**Figure 3:**
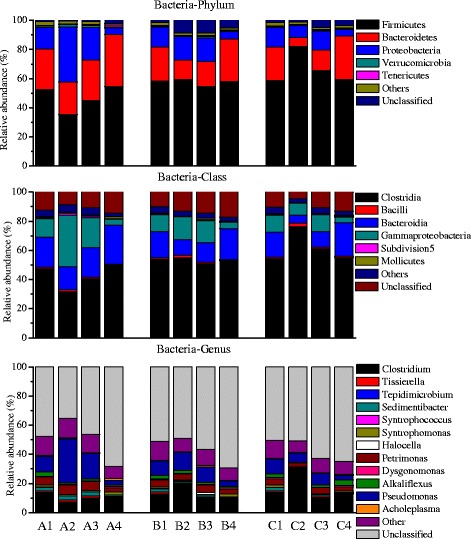
**Taxonomic classification of the bacteria communities.** Phyla, classes, and genera making up less than 1% of total composition in all the samples were classified as Others.

The numbers of sequences after quality filtration from different samples are shown in Additional file 1: Table S1. The average sequence lengths were around 273 bp for all the samples. The high-quality sequences were assigned to taxonomic classifications by the Ribosomal Database Project (RDP) classifier. Since the primers used in the present study were universal primers, sequences belonging to both bacteria and archaea were obtained at the same time [24,25]. The phylogenetic classification of sequences assigned to bacteria from all the samples is summarized in Figure 3. Samples A1, B1, and C1 had similar distributions of the sequences at the phylum, class, and genus levels. *Firmicutes*, *Bacteroidetes*, and *Proteobacteria* were dominant at the phylum level, and their dominance in biogas reactors was in accordance with other studies [26,27]. *Clostridia*, *Bacteroidia*, and *Gammaproteobacteria* were dominant at the class level. However, a considerable amount of the sequences (around 50%) were unclassified at the genus level, which could be due to some new microorganisms that have not yet been identified. The high percentages of unclassified sequences at the genus level were also found in previous studies [1,28]. The temperature disturbances had different effects on the shift of bacterial communities in reactors A, B, and C. The decrease of temperature from 37°C to 25°C in reactor A resulted in an increased abundance of *Proteobacteria* (A2), and the reason might be that some of the classes (for example, *Gammaproteobacteria*) belonging to *Proteobacteria* can grow well at lower temperatures [29]. In reactor B, the increase of temperature from 37°C to 45°C led to an increased relative abundance of the unclassified sequences at the phylum level (B2). The increased relative abundance of *Firmicutes* (C2) was observed with a further increase of temperature from 37°C to 55°C in reactor C. The relative abundance of class *Clostridia*, belonging to *Firmicutes*, was enriched in sample C2, which could be due to their spore-forming character and gradual adaptation to thermophilic conditions [26,30]. The bacterial communities (A3, B3, and C3, sampled on day 60) continued to change after the temperatures in all the reactors returned to 37°C, which was consistent with the unstable reactor performances (Figure 1, day 60). Samples A4, B4, and C4 were obtained during the steady states of phase III, and they had similar distributions at the phylum, class, and genus levels. However, compared with A1, B1, and C1, the relative abundance of *Bacteroidetes* in A4, B4, and C4 increased and that of *Proteobacteria* decreased. Differences at the class and genus levels were also observed between A1, B1, C1 and A4, B4, C4. The above results showed that although the biogas reactors, before and after the temperature disturbance, were run under exactly the same operational conditions, the bacterial communities did not return to the original bacterial composition. New steady-state bacterial community compositions, distinct from the original, were established after the temperature disturbances.

**Figure 4:**
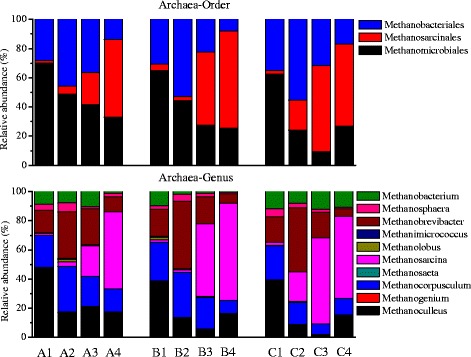
**Taxonomic classification of the archaea communities.**

The phylogenetic classification of sequences assigned to archaea from all the samples is summarized in Figure 4. The archaea mediating hydrogenotrophic and aceticlastic methanogenesis were found mainly within four orders (*Methanobacteriales*, *Methanococcales*, *Methanomicrobiales*, and *Methanosarcinales*) [31]. Therefore, only order- and genus-level classifications are shown in Figure 4. Samples A1, B1, and C1 were all dominated by *Methanomicrobiales* and *Methanobacteriales* in phase I, which belonged to hydrogenotrophic methanogens. Similar distributions of samples A1, B1, and C1 at the genus level were also observed, and the dominant genera were *Methanoculleus*, *Methanocorpusculum*, *Methanobrevibacter*, and *Methanobacterium*. An increase of *Methanobacterial*es was found in all the reactors after temperature disturbance, which may indicate that the archaea belonging to this order were more resistant to the temperature disturbance (both downwards and upwards). It has been reported that the most frequently observed hydrogen utilizers are members of *Methanobacteriales*, present in both manure and sewage sludge digesters [31]. Further study is needed in order to understand why *Methanobacteriales* were more resistant to temperature changes than other methanogens. In reactor C, the temperature increase resulted in the increased relative abundance of order *Methanosarcinales*, which are mainly aceticlastic methanogens. Since the methane production during temperature shock was significantly reduced, the changes of archaeal communities during the temperature disturbance might be due to the different decay rates of the archaea rather than the different growth rates of the archaea. After the temperature was changed back to 37°C, *Methanosarcinales* became dominant in all the reactors (A3, B3, and C3). At the steady states of phase III, *Methanomicrobiales*, *Methanosarcinales*, and *Methanobacteriales* were all dominant in reactors A, B, and C. The genus *Methanosarcina*, belonging to *Methanosarcinales*, mainly mediates aceticlastic methanogenesis, and therefore it is expected that the methanogenic pathway was changed before and after temperature disturbance. The dominance of *Methanosarcina* might be related to the better reactor performances in phase III compared with phase I. It has been reported that the dominance of hydrogenotrophic methanogenesis is always related to extreme conditions such as high ammonia or acetate concentration [32-34]. It is possible that the higher acetate concentration in phase I in all the reactors induced the dominance of archaea belonging to hydrogenotrophic methanogens.

**Figure 5:**
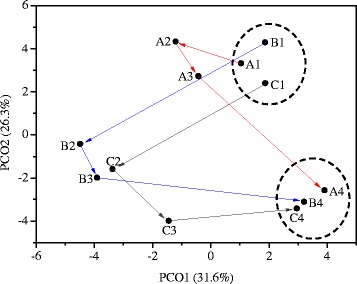
**PCoA of all the samples.**

The differences between the microbial communities of different samples were further assessed by principal coordinates analysis (PCoA) and hierarchical cluster analysis. The results from PCoA are shown in Figure 5. Principal components 1 and 2 explained 31.6% and 26.3% of the total community variations, respectively. A1, B1, and C1 were clustered together, and were well separated from the group of A4, B4, and C4. The results were consistent with the taxonomic analysis that steady-state microbial community compositions were changed after temperature disturbances in reactors A, B, and C, which could also be used to explain the different steady-state reactor performances in phase I and phase III, as discussed in the preceding section. A2 was close to A1, while B2 and C2 were far away from B1 and C1, which indicated that the increase of temperature to 45°C or 55°C in the biogas reactor induced significant changes in the microbial communities. A3, B3, and C3 were closer to A4, B4, and C4, which suggested that the microbial communities gradually changed to the new steady states after the temperature changed back to 37°C. The hierarchical cluster analysis (Additional file 1: Figure S1) also clustered A1, B1, and C1 as one group and A4, B4, and C4 as one group, which further supported the cluster results from PCoA.

### Microbial community assembly and function in anaerobic digestion of cattle manure

The results in the present study clearly showed that replicate biogas reactors treating cattle manure had similar steady-state reactor performances under the same environmental conditions. The replicate biogas reactors also had similar microbial community compositions based on RDP classification, PCoA, and hierarchical cluster analysis. However, Zhou *et al.* found that, under the same environmental conditions, both bacterial community compositions and functions in replicate microbial electrolysis cells were different, and they proposed that stochastic assembly played a dominant role in determining not only community structure but also ecosystem functions [35]. Based on our results, the microbial community compositions and functions in anaerobic digestion of cattle manure were not obviously affected by stochastic assembly. It seems that stochastic assembly played different roles in different ecosystems. A recent publication, which found that community history affects the predictability of microbial ecosystem development, might explain the differences between our results and Zhou’s results. Pagaling *et al.* [36] demonstrated that the final community composition and function are unpredictable when the source communities (inoculum) colonize a novel environment, while the community development is more reproducible when source communities are pre-conditioned to their new habitat. In our study, the inoculum was obtained from a biogas reactor, which was adapted to the anaerobic condition for methane production. Therefore, the steady-state microbial community composition and reactor performance were reproducible in the replicate biogas reactors. However, all the reactors in Zhou’s study were inoculated with wastewater not pre-conditioned in the microbial electrolysis cell reactors, which might have been the reason for the variation of microbial community compositions and functions observed. Further investigation is imperative to elucidate whether other inocula sources (sewage sludge, soil, and others), which are not derived from anaerobic reactors, would lead to different stable-state microbial communities and reactor performances in biogas reactors under the same operational conditions.

Although the effect of temperature disturbance on biogas production has been studied before [13,16], the previous studies only focused on the reactor performances. The rapid recovery of biogas production after the temperature disturbance was also previously observed [13], but in the cited study the biogas production reached the same level as that prior to the disturbance. We are the first to report the increased biogas production after temperature disturbance. The difference in biogas production recovery levels could be due to the fact that the substrate was already efficiently degraded before temperature shock in Chae’s study [13]. Our results suggested that temperature disturbance could be used as a strategy to stimulate biogas production in the biogas reactor, where the biogas production was not so efficient (possibly due to lower HRT). In a previous study, Nielsen *et al.* investigated the effects of disturbance of oleate on the performance of a biogas reactor treating cattle and pig manure, and they also found that a lower VFAs concentration along with a higher methane production were achieved in the biogas reactor after the disturbance of oleate [15]. However, they could not link the microbial community composition with the reactor performance due to the lack of microbial analysis. Our results combined with the above literature suggest that the idea that different types (temperature, oleate) of disturbances might have similar stimulation effects on the biogas process, and the effects of other disturbances (ammonia, organic loading shock, and others) on the community assembly and functioning of the biogas reactor deserve to be further investigated. Although in the present study, three different temperature disturbances were investigated, the establishment of new steady-state microbial community compositions in all the reactors after temperature disturbance was observed. More importantly, the three new steady-state microbial community compositions were clustered together and were clearly distinguished from the steady-state microbial community compositions before the temperature disturbances. A comparison of steady-state microbial community compositions before and after temperature disturbance showed that the temperature disturbance played an important role in the microbial community assembly and ecosystem function during the anaerobic digestion of cattle manure.

## Conclusions

The present study showed that similar steady-state process performances and microbial community profiles were achieved in three biogas reactors with the same inoculum and operational conditions, which suggested a minor role of stochastic factors in shaping the profile of the microbial community composition and activity in biogas reactor. Instead, temperature disturbance played an important role in the microbial community composition as well as process performance in biogas reactors. Increased methane yields (around 10% higher) and decreased VFAs concentrations at steady states were found in all three reactors after the temperature disturbances, although three different temperature disturbances were applied to each biogas reactor. New steady-state microbial community profiles were also observed in all the biogas reactors after the temperature disturbance.

## Materials and methods

### Inoculum and substrate

The inoculum (total solids (TS) 41.5 ± 1.4 g/L, VS 30.2 ± 1.2 g/L) used in this study was obtained from a mesophilic full-scale biogas plant (Hashoj biogas plant, Denmark) co-digesting cattle manure, pig manure, intestinal content from pig abattoirs, and fat and flotation sludge from pig abattoirs, fish and food processing industries, and so on. After collection, the inoculum was stored in an incubator with the temperature controlled at 37°C. The substrate used in the present study was cattle manure, and its characteristics were as follows: pH 7.5 ± 0.1, TS 36.6 ± 1.2 g/L, VS 25.2 ± 1.4 g/L, total nitrogen 2.2 ± 0.3 g/L.

### Reactor operation

Three identical 1000-mL continuously stirred tank reactors (A, B, and C) with working volumes of 700 mL were used. The reactors were mixed by magnetic stirrer at a stirring speed of 150 rpm, and the produced biogas was collected by gas bags. All the reactors were put into an incubator with the temperature controlled at 37°C. The HRT of the reactors was 14 d. Initially, all the reactors were filled with the above-mentioned inoculum (600 mL) and cattle manure (100 mL). After one week’s digestion, all the reactors were fed daily with cattle manure. Once steady states were achieved in all the reactors, the temperatures in the reactors were changed to 25°C, 45°C, and 55°C for reactors A, B, and C by putting them into different incubators with the corresponding temperatures, while the daily feeding with cattle manure continued. The temperatures were changed back to 37°C after 10 days’ operation period by putting all the reactors back into the initial incubator with the temperature controlled at 37°C, and all the reactors were operated with daily feeding at 37°C, until new steady states were achieved. A steady state in this study was defined as a stable biogas production with a daily variation of lower than 10% for at least 10 days.

### Microbial analysis

Four samples were collected from the mixture in each reactor for microbial analysis. Samples A1, B1, and C1 were collected during the steady states of reactors A, B, and C before the temperature disturbance (day 50). Samples A2, B2, and C2 were collected 10 days after the temperatures were changed to 25°C, 45°C, and 55°C for reactors A, B, and C, respectively (day 60). Samples A3, B3, and C3 were collected 10 days after the temperatures of all the reactors were changed back to 37°C (day 70). Samples A4, B4, and C4 were collected during the steady states of reactors A, B, and C after the temperature disturbance (day 112).

The total genomic DNA of the collected samples was extracted using QIAamp DNA Stool Mini Kit (51504, QIAGEN, Valencia, CA) according to the manufacturer’s instructions. The extracted DNA was amplified with the universal primers 515f and 806r [24,25]. The forward primer was 5’-GTGCCAGCMGCCGCGGTAA-3’, and the reverse primer was 5’-GGACTACHVGGGTWTCTAAT-3’. PCR conditions were set according to a previous study [24]. The PCR products were purified using the QIAquick spin columns (QIAGEN) to remove the excess primer dimers and dNTPs, and the concentration of PCR amplicons was measured by NanoDrop spectrophotometer [37]. The samples were then sent out for barcoded libraries preparation and sequencing on an Ion Torrent PGM system with 316 chip using the Ion Sequencing 400 bp Kit (all from Life Technologies) according to the standard protocol (Ion Xpress™ Plus gDNA and Amplicon Library Preparation, Life Technologies). The low-quality sequences without exact matches to the forward and reverse primers, with length shorter than 100 bp, and containing any ambiguous base calls, were removed from the raw sequencing data by RDP tools [38]. Chimeras were removed from the data by using the Find Chimeras web tool. The data were submitted to MG-RAST (ID 4556097.3-4556107.3). The sequences were phylogenetically assigned to taxonomic classifications by the RDP classifier with a confidence threshold of 50%. The PCoA and hierarchical cluster analysis were conducted by MG-RAST [39].

### Analytical methods

TS, VS, and total nitrogen were analyzed according to the American Public Health Association (APHA) [40]. The concentrations of acetate, propionate, isobutyrate, butyrate, iso-valerate, and valerate were determined by a gas chromatograph (Hewlett Packard, HP5890 series II) equipped with a flame ionization detector and HP FFAP column (30 m × 0.53 mm × 1.0 μm). CH_4_ was analyzed using a gas chromatograph with a thermal conductivity detector (GC-TCD) fitted with a parallel column of 1.1 m × 3/16 Molsieve 137 and 0.7 m × 1/4 Chromosorb 108. Detailed information about the operating conditions for the GC was given previously [41]. An analysis of variance was used to test the significance of results, and *P* < 0.05 was considered to be statistically significant. The methane yield was calculated based on the daily methane production (mLCH_4_/d) divided by daily VS feeding (gVS/d).

## Additional file

Additional file 1: Table S1. Number of high-quality sequences and the sequences assigned to bacteria and archaea based on RDP classification. **Figure S1.** Hierarchical cluster analysis of all the samples at the genus level.

## Abbreviations

AD: anaerobic digestion
HRT: hydraulic retention time
PCoA: principal coordinates analysis
TS: total solids
VFAs: volatile fatty acids
VS: volatile solids
