# Supplementary material for: New steady-state microbial community compositions and process performances in biogas reactors induced by temperature disturbances
Source: Biotechnol Biofuels. 2015 Jan 22;8:3. doi: 10.1186/s13068-014-0182-y (PMC4337199; doi:10.1186/s13068-014-0182-y)
Supplement: Additional file 1: Table S1. — Number of high-quality sequences and the sequences assigned to bacteria and archaea based on RDP classification. Figure S1. Hierarchical cluster analysis of all the samples at the genus level. [file 13068_2014_182_MOESM1_ESM.docx]

**Additional file**

New steady-state microbial community compositions and process performances in biogas reactors induced by temperature disturbances

Gang Luo^*1,2^, Davide De Francisci^2^, Panagiotis G. Kougias^2^, Treu Laura^2^, Xinyu Zhu^2^, Irini Angelidaki^2^

^1^ Shanghai Key Laboratory of Atmospheric Particle Pollution and Prevention (LAP3), Department of Environmental Science and Engineering, Fudan University, 200433, Shanghai, China

^2^Department of Environmental Engineering, Technical University of Denmark, DK-2800, Kgs Lyngby, Denmark

| Sample name | Total high quality sequences | Bacteria | Archaea |
| --- | --- | --- | --- |
| A1 | 77666 | 77277 | 389 |
| A2 | 109677 | 109238 | 439 |
| A3 | 108885 | 108449 | 436 |
| A4 | 102608 | 101479 | 1129 |
| B1 | 99381 | 98884 | 497 |
| B2 | 101229 | 100520 | 709 |
| B3 | 69379 | 68962 | 417 |
| B4 | 91529 | 90430 | 1099 |
| C1 | 116719 | 116252 | 467 |
| C2 | 109065 | 108628 | 437 |
| C3 | 89792 | 88983 | 809 |
| C4 | 103910 | 102559 | 1351 |

Table S1 Number of the high quality sequences and the sequences assigned to bacteria and archaea based on RDP classification


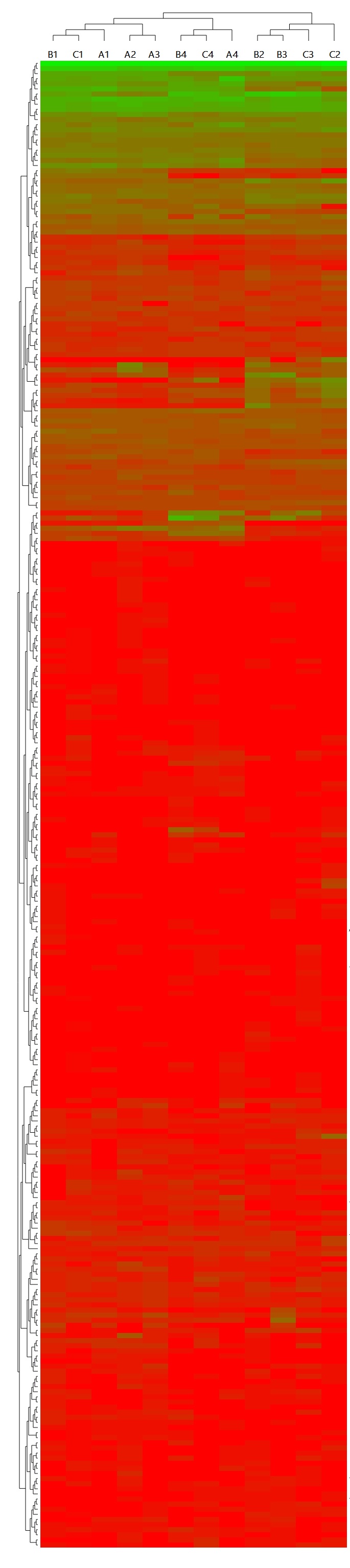


Fig S1 Hierarchical cluster analysis of all the samples in genus level.
